# Mental fatigue caused by prolonged cognitive load associated with sympathetic hyperactivity

**DOI:** 10.1186/1744-9081-7-17

**Published:** 2011-05-23

**Authors:** Kei Mizuno, Masaaki Tanaka, Kouzi Yamaguti, Osami Kajimoto, Hirohiko Kuratsune, Yasuyoshi Watanabe

**Affiliations:** 1 Molecular Probe Dynamics Laboratory, RIKEN Center for Molecular Imaging Science, 6-7-3 Minatojima-minamimachi, Chuo-ku, Kobe City, Hyogo 650-0047, Japan; 2 Department of Physiology, Osaka City University Graduate School of Medicine, 1-4-3 Asahimachi, Abeno-ku, Osaka City, Osaka 545-8585, Japan; 3 Department of Medical Science on Fatigue, Osaka City University Graduate School of Medicine, 1-4-3 Asahimachi, Abeno-ku, Osaka City, Osaka 545-8585, Japan; 4 Department of Health Science, Faculty of Health Science for Welfare, Kansai University of Welfare Sciences, 3-11-1 Asahigaoka, Kashihara City, Osaka 582-0026, Japan

## Abstract

**Background:**

It is known that chronic fatigue is associated with sympathetic hyperactivity. However, the relationship between autonomic function and mental fatigue caused by a prolonged mental load in healthy humans is still unclear. Thus, in order to clarify the mechanisms underlying mental fatigue, we examined the association between mental fatigue and autonomic functions.

**Methods:**

The study group comprised 10 healthy participants. To induce mental fatigue, participants performed mental tasks, which consisted of the advanced trail making test, *kana *pick-out test and mirror drawing test, for 8 hr, corresponding to a normal work day. Autonomic functions were measured by accelerated plethysmography before and after the fatigue-inducing mental tasks. As a control, the same participants completed an 8-hr relaxation session 4 weeks before the fatigue session.

**Results:**

After the 8-hr relaxation session, low-frequency component power (LF), high-frequency component power (HF) and low-frequency component power/high-frequency component power ratio (LF/HF ratio) were not changed from baseline. In contrast, after the fatigue session, the HF and LF/HF ratio were significantly changed from baseline; specifically, the HF was lower and LF/HF ratio was higher as compared to those after the relaxation session.

**Conclusions:**

Sympathetic hyperactivity based on decreased parasympathetic activity is associated with mental fatigue induced by prolonged cognitive load.

## Background

Fatigue, best defined as the difficulty in initiating or sustaining voluntary activities [1], is a common symptom of various illnesses, and can even be observed in healthy individuals [2-4]. Acute fatigue is a normal phenomenon that disappears after a period of rest. In contrast, long-term fatigue (chronic fatigue) is sometimes irreversible and the compensation mechanisms that are useful in reducing acute fatigue are no longer effective. Chronic fatigue is caused by the prolonged accumulation of acute fatigue. Thus, in order to avoid chronic fatigue, it is important to develop effective strategies to recover from and avoid the accumulation of acute fatigue.

Alterations of autonomic functions have been reported in patients with chronic fatigue syndrome (CFS) [5-10], multiple sclerosis (MS) [11-13], and primary biliary cirrhosis (PBC) [8,14]. In addition, autonomic dysfunction is associated with fatigue in patients with CFS [8], MS [11,12], and PBC [8,14]. These reports suggest that autonomic activities are related to the mechanisms underlying fatigue. However, this association has been demonstrated only in patients with specific diseases, not in healthy volunteers.

Fatigue is classified into physical and mental types. Physical fatigue, also known as peripheral fatigue, results from repeated muscle actions. In contrast, mental fatigue represents a failure to complete mental tasks that require self-motivation and internal cues in the absence of demonstrable cognitive failure or motor weakness [15]. Thus, mental fatigue decreases sufferers' work or study efficiency in daily life.

Recently, we demonstrated that decreased parasympathetic activity and increased sympathetic activity were induced in healthy volunteers following a 30-min series of fatigue-inducing mental tasks [16]. These results suggested that, as motivation has been reported to be associated with increased sympathetic activity [17,18], motivation against potential impairment of task performance caused by mental fatigue may contribute to the increased sympathetic activity observed during fatigue-inducing mental tasks. This finding clarified the relationship between mental fatigue and autonomic functions during fatigue-inducing mental tasks. However, the relationship between mental fatigue and autonomic functions during rest after the completion of a fatigue-inducing mental task remains unclear. Because deterioration of autonomic functions in patients with CFS, a human model of severe fatigue [19], is observed during rest, a difference in autonomic functions between chronic and acute fatigue might also be identified in healthy volunteers by evaluating autonomic functions during rest after a fatigue-inducing mental task. If the alternative pattern of autonomic functions is similar between CFS patients and fatigued healthy volunteers, the measurement of autonomic functions is useful as a common objective physiological marker for chronic and acute fatigue, as well as for evaluation of the effects of intervention against acute fatigue in order to prevent chronic fatigue. Therefore, the aim of the present study was to clarify the relationship between autonomic activity and mental fatigue after 8 hr of fatigue-inducing mental tasks, corresponding to a normal work day for most healthy adults.

## Methods

### Participants

The study group comprised 10 healthy volunteers [age, 27.6 ± 4.9 years (mean ± SD); 4 women, and 6 men]. Recruitment condition was decided based on previous studies [20-25]. The following potential study participants were excluded from the study: those who had a history of health problems; those taking chronic medication or supplemental vitamins; current smokers; those who weighed <40 kg; those who had donated blood within 1 month before the study; and those with a blood hemoglobin level <12.0 g/dl. Good health was also assessed by physical examination, blood pressure, heart rate, blood chemistry panel (glucose, creatinine, uremic nitrogen, sodium, potassium, chloride, uric acid, and creatine phosphokinase), lipid profile (total cholesterol and triglyceride), complete blood count, and urinalysis. Participants who manifested psychiatric morbidity (e.g., depression) were excluded from the study. Psychiatric evaluation and diagnosis were made by a psychiatrist (O. K.). The study protocol was approved by the Ethics Committee of Kansai University of Welfare Sciences and all participants provided written informed consent.

### Experimental design

All participants underwent both fatigue and relaxation sessions. The fatigue session was performed 4 weeks after the relaxation session in order to eliminate the effects of menstrual cycle for female participants. The day before either type of session, participants were instructed to avoid intensive physical and mental activities, to finish dinner by 10:00 p.m., and to fast overnight. At 9:00 a.m. the following morning, participants recorded their subjective sensation of fatigue on a visual analogue scale (VAS) from 0 (no fatigue) to 100 (complete exhaustion), and autonomic function was measured using accelerated plethysmography (APG). Participants then ate breakfast (carbohydrate, 73.6 g; protein, 26.9 g; lipid, 32.3 g; total calories, 707 kcal) between 9:45 and 10:15 a.m. After breakfast, either a relaxation or fatigue session was performed. During the fatigue sessions, participants performed an advanced trail making test [26,27] for 45 min, a *kana *pick-out test [28] for 30 min, and a mirror drawing test [29] for 45 min. Beginning at 10:15 a.m., four rotations of this series of tasks were performed for a total of 8 hr of mental fatigue-inducing activities. After the end of the second rotation, between 3:15 and 3:45 p.m., the participants ate lunch (carbohydrate, 124.2 g; protein, 26.4 g; lipid, 16.3 g; total calories, 844 kcal). The time interval between the first and second rotations and between the third and fourth rotations was 30 min. Upon completion of the fourth rotation, between 8:15 and 8:45 p.m., participants again recorded their subjective sensation of fatigue on the VAS, and autonomic functions were recorded using APG. During the relaxation sessions, participants read books, watched movies or talked among themselves for the same time frame used for the fatigue sessions.

### Fatigue-inducing mental tasks

The fatigue-inducing mental tasks used in the present study were chosen based on previous reports [21,22,25,27]. The advanced trail making test was an advanced version of the conventional trail making test [30]. In the test, circles numbered from 1 to 25 were randomly located on the display of a personal computer. Participants were required to touch the circles in sequence, starting with number 1. When participants clicked on the first target circle using the computer mouse, the circle disappeared and circle number 26 appeared in a different position on the screen. The positions of the other circles remained the same. Participants were required to memorize the positions of the other circles while searching for the target circle. Participants were instructed to complete the test as quickly and as accurately as possible.

In the *kana *pick-out test, participants were instructed to identify as many vowels as possible while at the same time understanding the meaning of the story in a Japanese-language novel. Every 8 min after the start of the test, participants were asked questions about the contents of the novel for 2 min in order to assess participants' reading comprehension.

In the mirror drawing test, participants traced a character on a small glass mirror, which reversed the image. Participants were instructed to perform this test as quickly and as accurately as possible.

### APG

Accelerated plethysmography has been used for the evaluation of autonomic functions [31-34]. In the present study, APG was performed using a pulsimeter (Artett, U-Medica, Osaka, Japan) with the sensor positioned on the tip of the ventral side of the index finger. Photoplethysmography was used to measure changes in the absorption of light by hemoglobin, which is related to blood flow volume. The pulsimeter performed automatic analyses of the second derivative of the photoplethysmographic waveform, which is known as the APG waveform. Participants underwent APG sitting quietly with their eyes closed for 2 min. The APG waveform consists of four waves in systole (a - d) and one in diastole (e). The sensor output of the pulsimeter was preprocessed by an analogue filter (2nd order, low pass filter with 23 Hz of cut off frequency). The data were recorded using an analogue-to-digital converter (3.3 volt to 10 bit) and a real-time sampling rate of 1,000 samples per second. These digital data were processed with a 67th order finite impulse response filter using the Hanning window. Detected peak times were interpolated to sub-millisecond order. Frequency analyses for pulse-interval variation were analyzed with fast Fourier transform. Resolution ability for power spectrum was 0.001 Hz. For the frequency analyses, the total power was calculated as the power within a frequency range of 0 - 0.4 Hz, the low-frequency component power (LF) was calculated as the power within a frequency range of 0.04 - 0.15 Hz, and the high-frequency component power (HF) was calculated as that within a frequency range of 0.15 - 0.4 Hz. The HF is vagally mediated [35-37], whereas the LF originates from a variety of sympathetic and vagal mechanisms [35,38]. The low-frequency component power/high-frequency component power ratio (LF/HF ratio) is considered to represent sympathetic activity [39].

### Statistical analyses

The VAS values for fatigue and pulse rates are shown as the mean ± SD. When statistically significant effects for session (relaxation and fatigue) or time course (baseline and 8 hr) and/or session × time course interactions in each value were found by two-way repeated measures analysis of variance (ANOVA), and a paired *t*-test was used to evaluate the significance of differences between the relaxation and fatigue sessions or between baseline and 8 hr for each session. A Wilcoxon signed-rank test was used to evaluate the significance of differences in each spectral component between the relaxation and fatigue sessions or between baseline and 8 hr for each session. Spearman's and Pearson's correlation analyses between the VAS value for fatigue and LF/HF ratio and log-transformed LF/HF ratio (ln LF/HF ratio) were performed, respectively. All *p *values were 2-tailed, and *p *values <.05 were considered statistically significant. Statistical analyses were performed using the SPSS 17.0 software package (SPSS Inc., Chicago, IL).

## Results

Visual analogue scale values for fatigue and pulse rates before and after the relaxation and fatigue sessions are shown in Table 1. Although two-way repeated measures ANOVA of the VAS values for fatigue revealed no significant effect of session (*F*_(1,9) _= 2.03, *p *= .188), it revealed a significant effect of time course (*F*_(1,9) _= 7.50, *p *= .023) and session × time course interactions (*F*_(1,9) _= 12.74, *p *= .006). The VAS values for fatigue before the experiment (baseline) did not differ between the relaxation and fatigue sessions. The VAS value for fatigue was increased from baseline after the fatigue session, and the VAS value for fatigue after the fatigue session was greater than that after the relaxation session, indicating that the series of mental tasks performed during the fatigue session induced fatigue in the participants. Two-way repeated measures ANOVA of pulse rates revealed no significant effect of session (*F*_(1,9) _= 3.10, *p *= .112), time course (*F*_(1,9) _= 3.60, *p *= .090) or session × time course interactions (*F*_(1,9) _= 2.28, *p *= .165).

**Table 1:** Visual analogue scale (VAS) value for fatigue and autonomic activities before (Baseline) and after (8 hr) the relaxation and fatigue sessions

	Relaxation session		Fatigue session	
	
	Baseline	8 hr	Baseline	8 hr
VAS value for fatigue	41.0 ± 19.8	45.4 ± 20.4	31.8 ± 21.3	70.5 ± 20.6**, *^†^
Accelerated plethysmography				
Pulse rate (bpm)	75.9 ± 21.6	66.9 ± 9.8	68.9 ± 12.3	65.5 ± 9.25
Total power (ms^2^)	2297 (1445 - 3209)	2116 (1407 - 3087)	2849 (1776 - 4019)	1300 (1160 - 2877)**
LF (ms^2^)	825 (501 - 1234)	745 (430 - 1137)	758 (447 - 1338)	946 (794 - 1829)
HF (ms^2^)	1074 (654 - 1481)	806 (571 - 1049)	1304 (739 - 1880)	324 (219 - 602)**, ^††^
LF/HF ratio	0.75 (0.59 - 1.72)	0.97 (0.53 - 1.55)	0.56 (0.39 - 1.67)	2.92 (2.44 - 3.69)**, ^†^

LF, low-frequency component power; HF, high-frequency component power; LF/HF ratio, low-frequency component power/high-frequency component power ratio. Values are presented as the mean and SD or median and inter-quartile range. **p < .01, significantly different from the corresponding values for the Baseline in fatigue session (paired *t*-test or Wilcoxon signed-rank test). ^†^*p *< .05, ^††^*p *< .01, significantly different from the corresponding values for the 8 hr in relaxation session (paired *t*-test or Wilcoxon signed-rank test)

Autonomic functions before and after the relaxation and fatigue sessions are shown in Table 1. The total power at baseline did not differ between the relaxation and fatigue sessions. Although the total power was not altered from the baseline after the relaxation session, the total power was decreased from the baseline after the fatigue session. Low-frequency component power between the relaxation and fatigue sessions or between the baseline and 8 hr for each session were similar. High-frequency component power at baseline did not differ between the relaxation and fatigue sessions. The HF was decreased from the baseline after the fatigue session, and the HF after the fatigue session was lower than that after the relaxation session. Although the LF/HF ratio at baseline did not differ between the relaxation and fatigue sessions, the LF/HF ratio was increased from baseline after the fatigue session, and the LF/HF ratio after the fatigue session was higher than that after the relaxation session.

**Figure 1:**
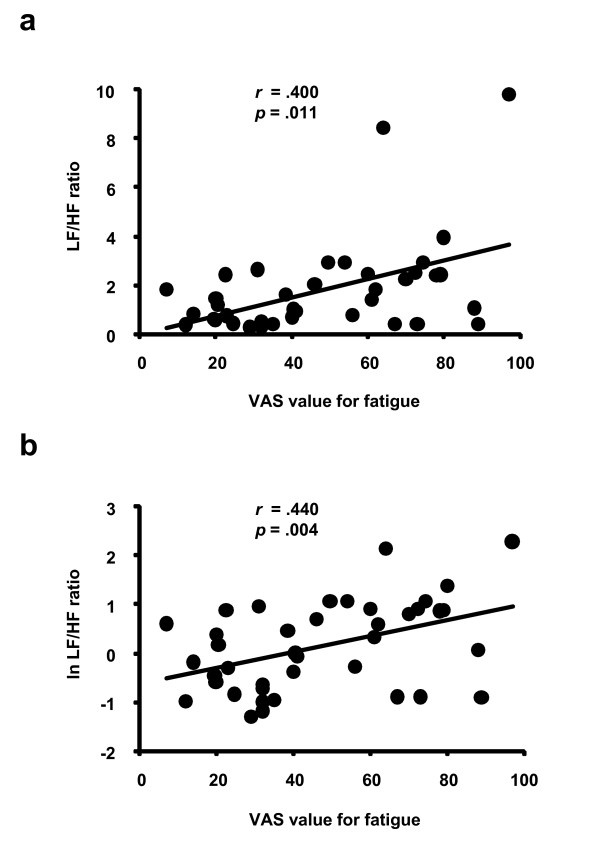
**Correlations between visual analogue scale (VAS) value for fatigue and autonomic activities**. Low-frequency component power/high-frequency component power ratio (LF/HF ratio; a) log-transformed LF/HF ratio (ln LF/HF ratio; b) obtained on a wave interval analyses using accelerated plethysmography in all the measurement points before and after the 8-hr fatigue and relaxation sessions. Spearman's (a) and Pearson's (b) correlation coefficients and *p*-values are shown.

Correlation analyses between the VAS value for fatigue and the LF/HF ratio or the ln LF/HF ratio in all the measurement points which are before and after the 8-hr fatigue and relaxation sessions are shown in Figure 1. The VAS values for fatigue were positively correlated with the LF/HF ratio (Figure 1a) and the ln LF/HF ratio (Figure 1b).

## Discussion

The present study showed that, whereas the HF and the LF/HF ratio did not change from baseline after the relaxation session, these values changed significantly after the fatigue session. After the 8-hr fatigue session, the HF was lower and the LF/HF ratio was higher than those after the 8-hr relaxation session. In addition, levels of fatigue sensation were associated with the LF/HF ratio.

The present results suggest that parasympathetic activity was reduced by mental fatigue. Previous studies have consistently demonstrated a decrease in parasympathetic activity due to mental load [40-45]. These findings suggest that mental load causes reduced parasympathetic activity, and that prolonged fatigue-inducing mental load reduces parasympathetic activity even further. Thus, reduced parasympathetic activity seems to be a characteristic feature of mental fatigue.

Recently, we demonstrated that not only decreased parasympathetic activity and but also increased sympathetic activity were induced in healthy volunteers following a 30-min series of fatigue-inducing mental tasks [16], suggesting that, because motivation has been reported to be associated with increased sympathetic activity [17,18], motivation against potential impairment of task performance caused by mental fatigue may contribute to the increased sympathetic activity observed during fatigue-inducing mental tasks. However, in the present study, sympathetic activity based on decreased parasympathetic activity was also observed despite the allotment of a rest period after the fatigue-inducing mental tasks. These results suggest that decreased parasympathetic activity and increased sympathetic activity induced by mental fatigue are associated with not only motivation but also other components.

Decreased parasympathetic activity and increased sympathetic activity are interpreted as a state of autonomic hypervigilance [46,47]. In order to enhance parasympathetic responses for vegetative and restorative functions, and more generally under conditions of normal modern life, sympathoexcitatory response needs to be tonically inhibited [46]. Accordingly, sympathoexcitatory subcortical threat circuits are normally under the inhibitory control of the prefrontal cortex [46,48,49]. Several studies have reported that during prolonged mental tasks lasting 1 - 2 hr, activation of the brain regions relating to mental task processing were gradually reduced [50,51]. During the fatigue-inducing mental tasks in the present study, the prefrontal cortex, which is associated with the processing of executive functions such as visuospatial working memory and divided attention in the advanced trail making test and *kana *pick-out test, respectively, may have been continuously activated. Therefore, although further neuroimaging studies are necessary, prolonged cognitive load may induce decreases in prefrontal cortex activity and inhibitory capacity for sympathoexcitatory response.

Consistent with our results, decreased parasympathetic activity and increased relative sympathetic activity have been observed in CFS patients [9,10,31]. Evidence such as lowered cerebral activity in the prefrontal cortex during fatigue-inducing tasks [52] and a bilateral reduction of grey-matter volume in the prefrontal cortices [53] suggests that individuals with CFS may exhibit anatomical and functional alterations in the prefrontal cortex. Since the role of the prefrontal cortex is essential in active tonic inhibition of sympathoexcitatory threat circuits, such alterations in the prefrontal cortex seen in CFS patients could be expected to lead to a decrease in parasympathetic drive, defaulting to a sympathetically driven system. Therefore, it is possible that an accumulation of mental fatigue in healthy people induces a prolonged deterioration of autonomic activity through anatomical and functional alterations of the prefrontal cortex. In order to prevent healthy individuals from suffering from chronic fatigue, early intervention and evaluation of the effect of intervention are very important. For this purpose, autonomic functions may be useful as objective physiological markers for acute and chronic fatigue.

## Limitations

The present study has limitations. The standard guidelines for analyses of heart rate variability noted that LF and HF spectral components are distinguished in a spectrum calculated from short-term recordings of 2 to 5 min [54] and the study in this field has been performed based on the standard guidelines [55]. In the present study, since the APG was recorded for 2 min, the length of recording time was insufficient and the effect of breathing was not excluded adequately [54]. The number of participants tested in the present study was limited. In order to generalize our results, studies involving a larger number of participants with recordings over longer time intervals are essential.

## Conclusions

The present results provide evidence that decreased parasympathetic activity and increased relative sympathetic activity are associated with mental fatigue induced by prolonged cognitive load in healthy adults. Consistent with the present findings, alterations of autonomic functions, such as decreased parasympathetic activity and increased relative sympathetic activity, have been reported in patients with CFS [9,10,31]. Therefore, it is possible that mental fatigue can be induced by daily events in healthy people and may eventually progress to chronic fatigue. In contrast to acute fatigue, which is a physiological phenomenon that disappears after a period of rest, chronic fatigue is sometimes irreversible, and the compensation mechanisms useful in reducing acute fatigue are not effective for chronic fatigue [56]. Thus, it is important to identify the risk factors for chronic fatigue. Our findings provide new perspectives on the mechanisms underlying acute and chronic fatigue.

## List of abbreviations

ANOVA: Analysis of variance; APG: Accelerated plethysmography; CFS: Chronic fatigue syndrome; HF: High-frequency component power; LF: Low-frequency component power; LF/HF ratio: Low-frequency component power/high-frequency component power ratio; ln LF/HF ratio: Log-transformed LF/HF ratio; MS: Multiple sclerosis; PBC: Primary biliary cirrhosis; SD: Standard deviation; VAS: Visual analogue scale.

## Competing interests

The authors declare that they have no competing interests.

## Authors' contributions

KM took part in designing and planning the experiment, data analyses and manuscript preparation. MT contributed to designing and planning the experiment, data analyses and manuscript preparation. KY contributed to designing and planning the experiment and analysing the data. OK and HK contributed to designing and planning the experiment. YW took part in planning and designing the experiment and preparing the manuscript. All authors read and approved the final manuscript.
